# Body mass index and clinical outcomes in individuals with major depressive disorder: Findings from the GSRD European Multicenter Database

**DOI:** 10.1016/j.jad.2023.05.042

**Published:** 2023-05-16

**Authors:** Christoph Kraus, Alexander Kautzky, Victoria Watzal, Anna Gramser, Bashkim Kadriu, Zhi-De Deng, Lucie Bartova, Carlos A. Zarate, Rupert Lanzenberger, Daniel Souery, Stuart Montgomery, Julien Mendlewicz, Joseph Zohar, Giuseppe Fanelli, Alessandro Serretti, Siegfried Kasper

**Affiliations:** a Department of Psychiatry and Psychotherapy, Medical University of Vienna, Vienna, Austria; b Experimental Therapeutics and Pathophysiology Branch, National Institute of Mental Health, National Institutes of Health, Bethesda, MD, USA; c Comprehensive Center for Clinical Neurosciences and Mental Health, Medical University of Vienna; d Laboratoire de Psychologie Medicale, Université Libre de Bruxelles and Psy Pluriel at Epsylon Caring for Mental Health Brussels, Brussels, Belgium; e Imperial College School of Medicine, London, United Kingdom; f Université Libre de Bruxelles, Brussels, Belgium; g Department of Psychiatry, Sheba Medical Center, Tel Hashomer, Sackler School of Medicine, Tel Aviv University, Israel; h Department of Biomedical and NeuroMotor Sciences, University of Bologna, Italy; i Department of Human Genetics, Radboud University Medical Center, Donders Institute for Brain, Cognition and Behaviour, Nijmegen, the Netherlands; j Center for Brain Research, Department of Molecular Neuroscience, Medical University of Vienna, Vienna, Austria

**Keywords:** Major depression, Comorbidity, Insulin resistance, Cognition, Treatment-resistant depression

## Abstract

**Background:**

Individuals with major depressive disorder (MDD) are at higher risk for obesity. In turn, weight gain is a predisposing factor for depression. Although clinical data are sparse, suicide risk also appears to be elevated in obese patients. This study used data from the European Group for the Study of Resistant Depression (GSRD) to investigate clinical outcomes associated with body mass index (BMI) in MDD.

**Methods:**

Data were drawn from 892 participants with MDD over the age of 18 years (580 female, 50.5 ± 13.6 years). Response and resistance to antidepressant medication, depression rating scale scores, and further clinical and sociodemographic variables were compared using multiple logistic and linear regressions controlled for age, sex, and risk of weight gain due to psychopharmacotherapy.

**Results:**

Of the 892 participants, 323 were categorized as treatment-responsive and 569 as treatment-resistant. Within this cohort, 278 (31.1 %) were overweight (BMI = 25–29.9 kg/m^2^) and 151 (16.9 %) were obese (BMI > 30 kg/m^2^). Elevated BMI was significantly associated with higher suicidality, longer duration of psychiatric hospitalizations over their lifetimes, earlier age of onset of MDD, and comorbidities. There was a trend-wise association of BMI with treatment resistance.

**Limitations:**

Data were analyzed in a retrospective, cross-sectional design. BMI was used as an exclusive measure of overweight and obesity.

**Conclusions:**

Participants with comorbid MDD and overweight/obesity were at risk for worse clinical outcomes, suggesting that weight gain should be closely monitored in individuals with MDD in daily clinical practice. Further studies are needed to explore the neurobiological mechanisms linking elevated BMI to impaired brain health.

## 1. Introduction

The World Health Organization estimates that about 1.9 billion adults were overweight or obese in 2016, a major health hazard associated with a number of non-communicable diseases such as type 2 diabetes mellitus and cardiovascular disease. In addition to other somatic comorbidities, existing research suggests that overweight and obesity play a role in the etiopathogenesis of major depressive disorder (MDD) (Fanelli and Serretti, 2022; Mansur et al., 2015; Milaneschi et al., 2019). In this regard, a positive genetic correlation was found between MDD and body mass index (BMI) and obesity, indicating the existence of a shared genetic risk and common biological mechanisms (Fanelli et al., 2022). Moreover, a Mendelian randomization study highlighted a causal role of BMI on depression, while not ruling out a possible two-way causal relationship between the two, as suggested by previous studies (Luppino et al., 2010; Tyrrell et al., 2019). For instance, a previous meta-analysis of longitudinal studies found a bidirectional association between clinical depression and obesity, suggesting that depression aggravates obesity and vice versa (Luppino et al., 2010). Interestingly, a nonlinear exposure–response pattern has also been suggested, wherein being overweight prevented depression in men compared to being underweight (BMI < 18.5) or obese (BMI > 30) (Mulugeta et al., 2019). In line with these findings, studies similarly found a U-shaped association between BMI and depression, with higher depression scores in underweight and obese patients (de Wit et al., 2009; Jung et al., 2017). In addition, Silva and colleagues identified anthropometric profiles that differed across depression subtypes; specifically, the symptomatology associated with atypical depression was related to BMI, whereas the symptomatology associated with melancholic depression was not (Silva et al., 2020). Using different methods, Wiltink and colleagues found that somatic (i.e., psychomotor agitation/retardation, sleep, fatigability, and appetite) but not cognitive-affective dimensions of depression were associated with measures of obesity (Wiltink et al., 2013).

The relationship between suicidal behavior and BMI suggests an inverse correlation between BMI and suicide death, with obesity being associated with an increased risk of attempted suicide and lower body weight with higher rates of completed suicide (Klinitzke et al., 2013; Mukamal et al., 2009; Perera et al., 2016; Zhang et al., 2013). Another study found a positive link between BMI and suicidal ideation (Dutton et al., 2013), but this was not confirmed in a systematic review by Perera and colleagues (Perera et al., 2016).

To date, evidence of the impact of BMI on the clinical course of illness in depressed individuals has been scarce. Previous clinical studies indicated that obesity or overweight put individuals with depression at increased risk for insufficient response to antidepressant medications or treatment resistance (Puzhko et al., 2020; Uher et al., 2009). Similarly, our previous results from a multivariate prediction model found that BMI was among the most influential predictors of treatment resistance (Kautzky et al., 2018). However, little evidence exists regarding which clinical, sociodemographic, or outcome variables are influenced by BMI in individuals with MDD. This retrospective analysis was performed using the multicenter database of the European Group for the Study of Resistant Depression (GSRD) (Bartova et al., 2019; Dold et al., 2018; Dold et al., 2016; Fugger et al., 2018; Schosser et al., 2012). The primary aim was to investigate the impact of BMI on patient outcomes in MDD, and the primary hypothesis was that higher BMI would negatively impact treatment outcomes, possibly leading to increased resistance to antidepressants.

## 2. Methods

### 2.1. Study sample

Both treatment-responsive and treatment-resistant participants for this retrospective analysis were drawn from an ongoing multi-center clinical database study conducted by the GSRD (Bartova et al., 2019). Each local investigational review board approved the study protocol and all study-related procedures, and each patient provided written informed consent after being provided with oral and written information on study procedures. The GSRD recruited individuals at 10 academic sites across Europe and Israel between 2011 and 2017 (Austria, Belgium, France (two sites), Germany, Greece, Israel, Italy (two sites), and Switzerland); participants could be treated as inpatients or outpatients depending on the site. All participants had received a current and primary diagnosis of MDD, were over the age of 18, and had been treated with at least one antidepressant agent at sufficient duration and dose during their current major depressive episode (MDE); the antidepressant trial was defined as adequate if it lasted at least four weeks and if the dose was equal to or higher than the lowest dose defined as effective according to international treatment recommendations (Dold and Kasper, 2017; Souery et al., 2007). Patient records were screened for inclusion by clinical and research psychiatrists at the relevant academic centers, and participants diagnosed with bipolar disorder, schizoaffective disorder, any severe substance abuse disorder (excluding nicotine and caffeine), or severe comorbid personality disorder based on clinical judgement were excluded.

All participants were screened by clinically experienced psychiatrists; structured diagnostic interviews were conducted using the MINI-Interview (Sheehan et al., 1998). All eligible participants were evaluated and rated by trained clinical psychiatrists, scales for affective disorders were performed (see below), and biometric, clinical, and sociodemographic variables were collected.

### 2.2. Measures of clinical obesity

Weight and height were collected from the medical records of the included participants; these were either measured or self-reported. BMI was calculated as weight in kilograms divided by squared height in meters and used as both a categorical and continuous variable in this study. The following standardized BMI categories established by the World Health Organization were used for categorization purposes: “underweight” (BMI < 18.5); “below normal weight” (BMI ≥ 18.5 to <20); “normal weight” (BMI ≥20–25); “overweight” (BMI ≥25 to <30); “class I obesity” (BMI ≥30 to <35); “class II obesity” (BMI ≥35 to <40); “class III obesity or extreme or severe obesity” (BMI ≥40) (NCD-RisC, 2016).

### 2.3. Clinical outcomes

At the baseline visit, the standardized neuropsychological battery included the Montgomery–Åsberg Depression Rating Scale (MADRS) (Montgomery and Asberg, 1979). In addition, the severity at onset of the present MDE was rated retrospectively using the MADRS as well as information drawn from the clinical interview and medical records. This procedure allowed us to estimate symptom improvement over the course of the current MDE by subtracting the retrospective MADRS (rMADRS) score before treatment from the current MADRS (cMADRS) score post-treatment (Bartova et al., 2019). While this approach has limitations with regard to recall bias, it allows for the inclusion of participants at a single time point, thus increasing the likelihood of inclusion.

Antidepressant treatment across centers is detailed in a previous publication of the GSRD database (Dold et al., 2016). Response to antidepressant psychopharmacotherapy administered during the current MDE was defined as a ≥50 % reduction of the MADRS. Treatment resistance was defined as non-response to at least two or more consecutive antidepressant trials of sufficient dose and duration that might have included further combination and augmentation treatment strategies, in line with recent clinical trials in treatment-resistant depression (Daly et al., 2018). Suicidality, including suicidal intent and behavior, was assessed as a dichotomous item for lifetime suicidality in the database (binary yes/no) as well as via item 10 from the MADRS (7-item scale, 0–6).

### 2.4. Statistical analyses

**Fig. 1.**
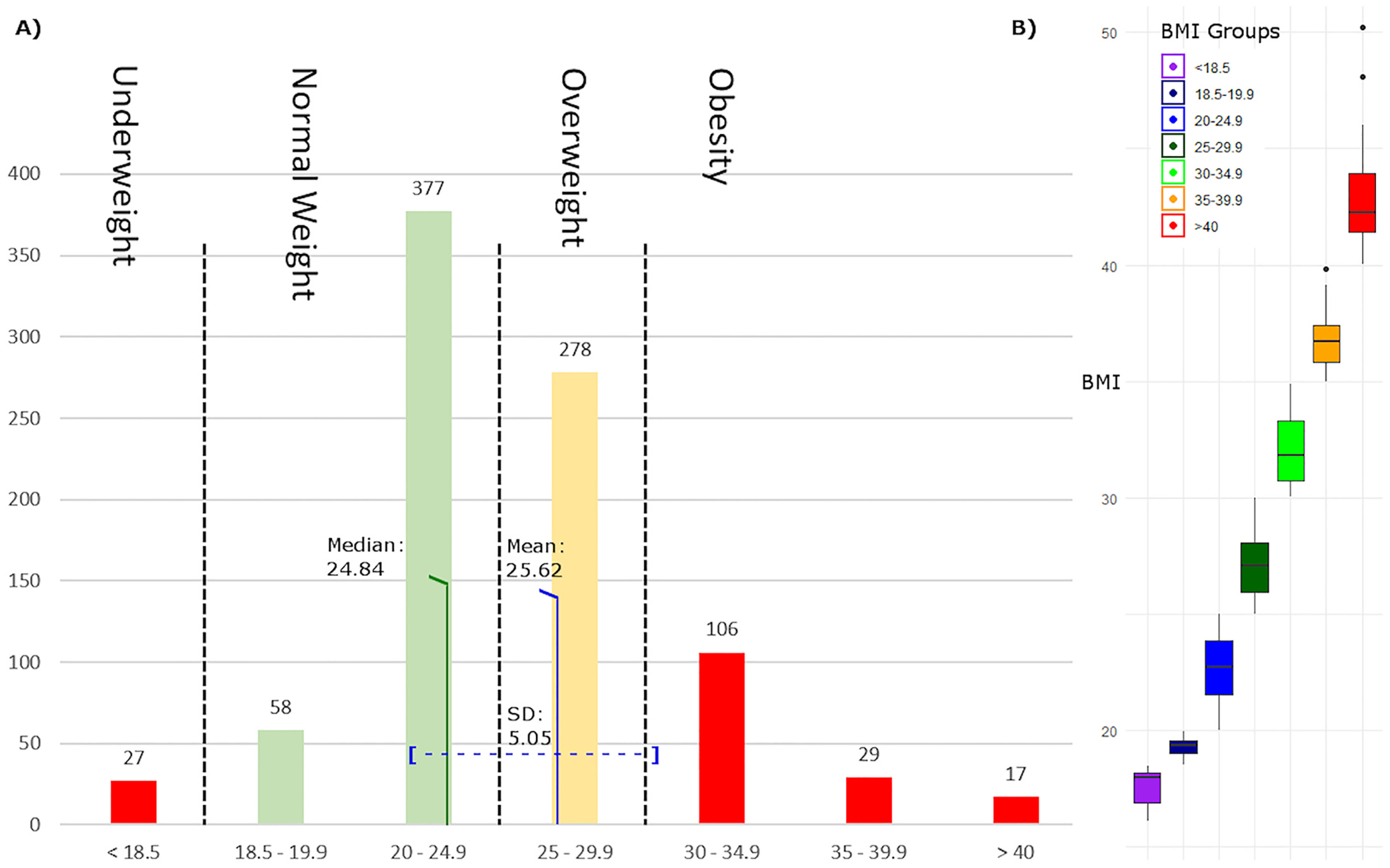
A) Body mass index (BMI) distribution and number of individuals in each BMI category. Mean and median indicate BMI values of the overall sample. B) Boxplots of the BMI categories.

All statistical analyses were conducted in R version 4.0.4 (www.r-project.org). Sample size characteristics and BMI categories are presented with frequentist statistics in Fig. 1. Logistic regression with ‘yes/no’ variables as dependent variables and BMI (metric) as an independent variable was used to assess whether BMI affected treatment response status as well as psychiatric comorbidities and other binomial clinical and psychosocial parameters (see Table 1). Linear regression with BMI (metric) as an independent variable was used for analysis of continuous outcome variables, including baseline and post-treatment MADRS items (see Table 1). All regression models further included age and sex as well as a variable representing the risk of weight gain caused by any current psychopharmacotherapy. Towards this end, an individual ‘risk score’ was calculated for each patient: +1 for medications associated with weight gain, 0 for medications that did not influence weight, and − 1 for medications associated with weight loss (Serretti and Mandelli, 2010). Uncorrected p-values are reported with a significance level of two-tailed alpha <0.05. Results that withstood Bonferroni correction are highlighted in Table 1 (corrected p-threshold 0.0006).

#### 3. Results

In total, 892 participants with MDD (580 female, 50.5 ± 13.6 years) were categorized as either treatment responders (n = 323, 36.2 %) or treatment-resistant (n = 569, 63.8 %). Within this cohort, 278 (31.1 %) of all participants were overweight (BMI = 25–29.9 kg/m^2^), and an additional 151 (16.9 %) were obese (BMI > 30 kg/m^2^); thus approximately 48 % of the total sample was either overweight or obese. Mean BMI was 25.6 kg/m^2^ (Fig. 1).

As shown in Table 1, higher BMI was significantly associated with presence of suicidality (binary yes/no item) (F(1, 887) = 9.42, p = 0.002), longer duration of psychiatric hospitalizations over their lifetimes (F(1, 852) = 7.71, p = 0.006), and earlier onset of first MDE (F(1, 852) = 6.49, p = 0.029). There was also a trend towards higher BMI in treatment-resistant participants (F(1, 887) = 3.01, p = 0.085) and a trend towards more obese individuals exhibiting a greater number of previous MDEs (F(1, 712) = 3.07, p = 0.08).

A significant association was also observed between higher psychopharmacotherapy-related weight gain risk score and resistance to antidepressants (F(1, 887) = 21.44, p < 0.0001). However, no significant association was found between length of the current MDE and BMI (p > 0.05).

With regard to comorbidities, higher BMI was significantly associated with an increased incidence of comorbid agoraphobia (t = 2.73, p = 0.006), social phobia (t = 2.103, p = 0.035), and post-traumatic stress disorder (PTSD) (t = 3.14, p = 0.002). No association was observed between BMI and generalized anxiety disorder, panic disorder, obsessive-compulsive disorder, psychotic features, or dysthymia (all yes/no items, p > 0.05).

**Fig. 2.**
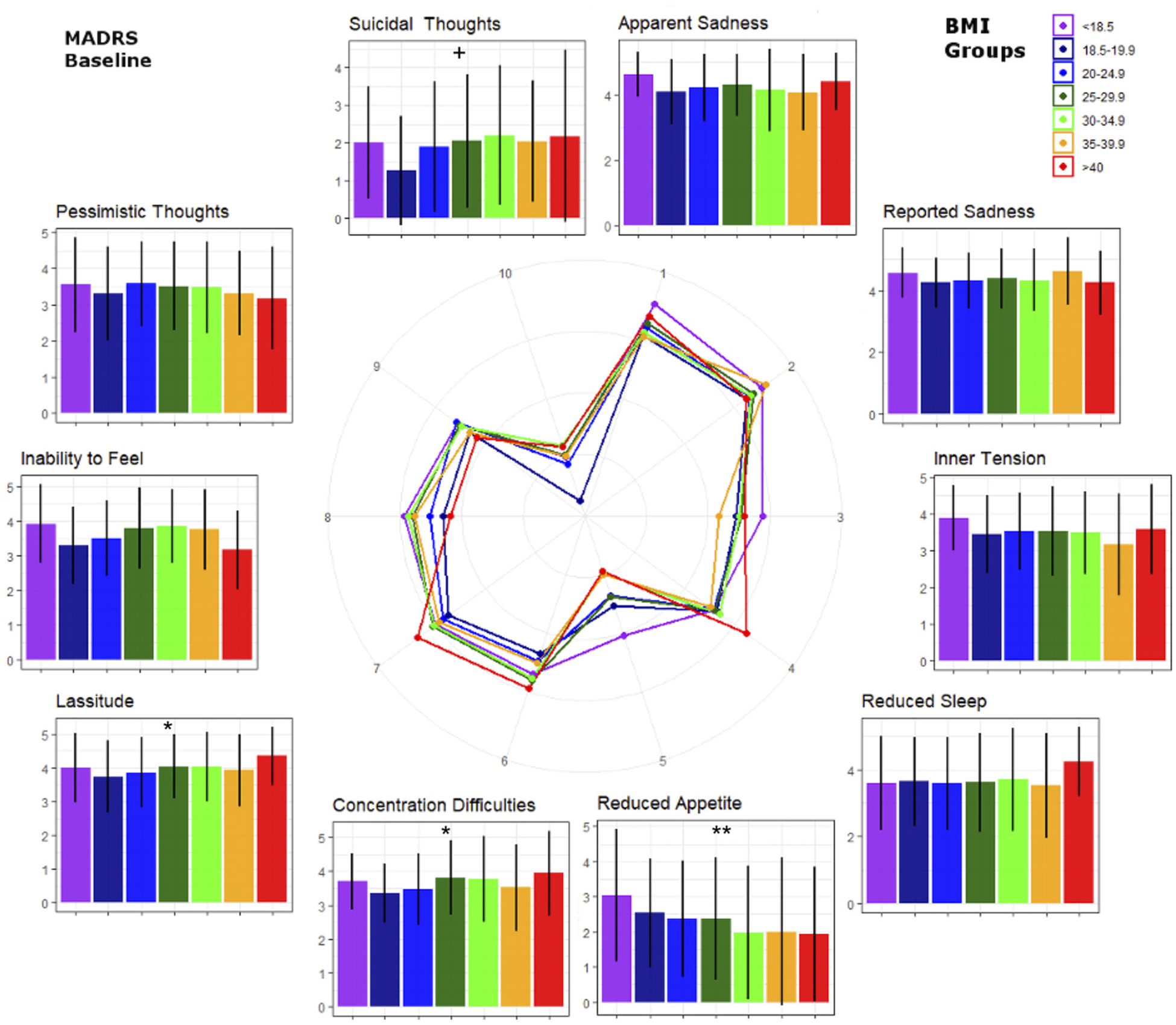
Retrospective Montgomery–Åsberg Depression Rating Scale (MADRS) sub-item scores by body mass index (BMI) groups (i.e., baseline BMI values). Numbers in the spider graph represent MADRS subitem scales from 0 to 6. BMI groups correspond to those in Fig. 1. *p < 0.05 uncorrected, **Bonferroni corrected p-values. ^+^Trend-wise significance (p < 0.1).

**Fig. 3.**
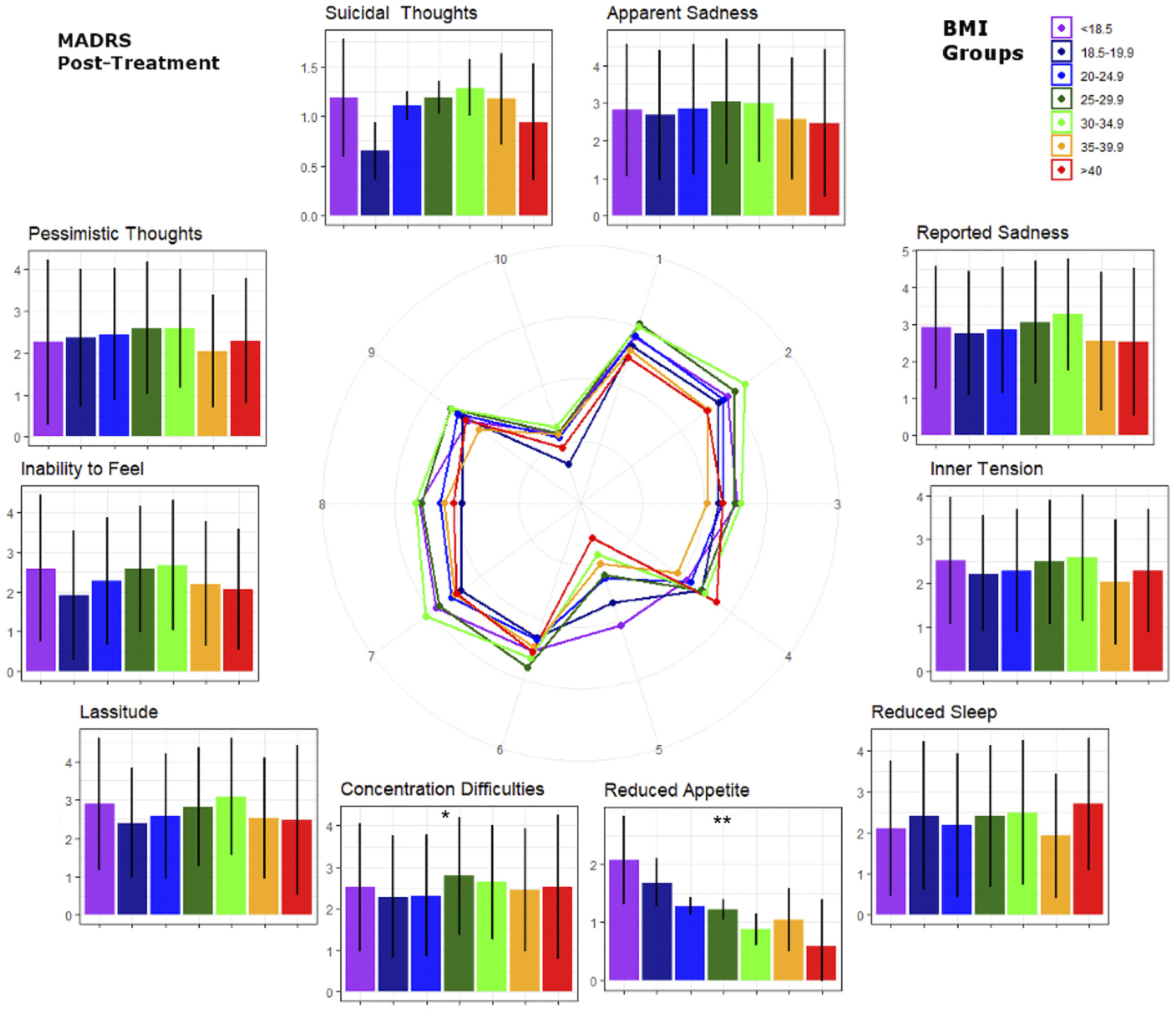
Post-treatment Montgomery–Åsberg Depression Rating Scale (MADRS) sub-item scores by body mass index (BMI) groups (i.e., post-treatment BMI values). BMI groups correspond to those in Fig. 1. Numbers in the spider graph represent MADRS subitem scales from 0 to 6. Note differences from baseline values in the ‘lassitude’ and ‘suicidal thoughts’ items. *p < 0.05 uncorrected, **Bonferroni corrected p-values.

**Fig. 4.**
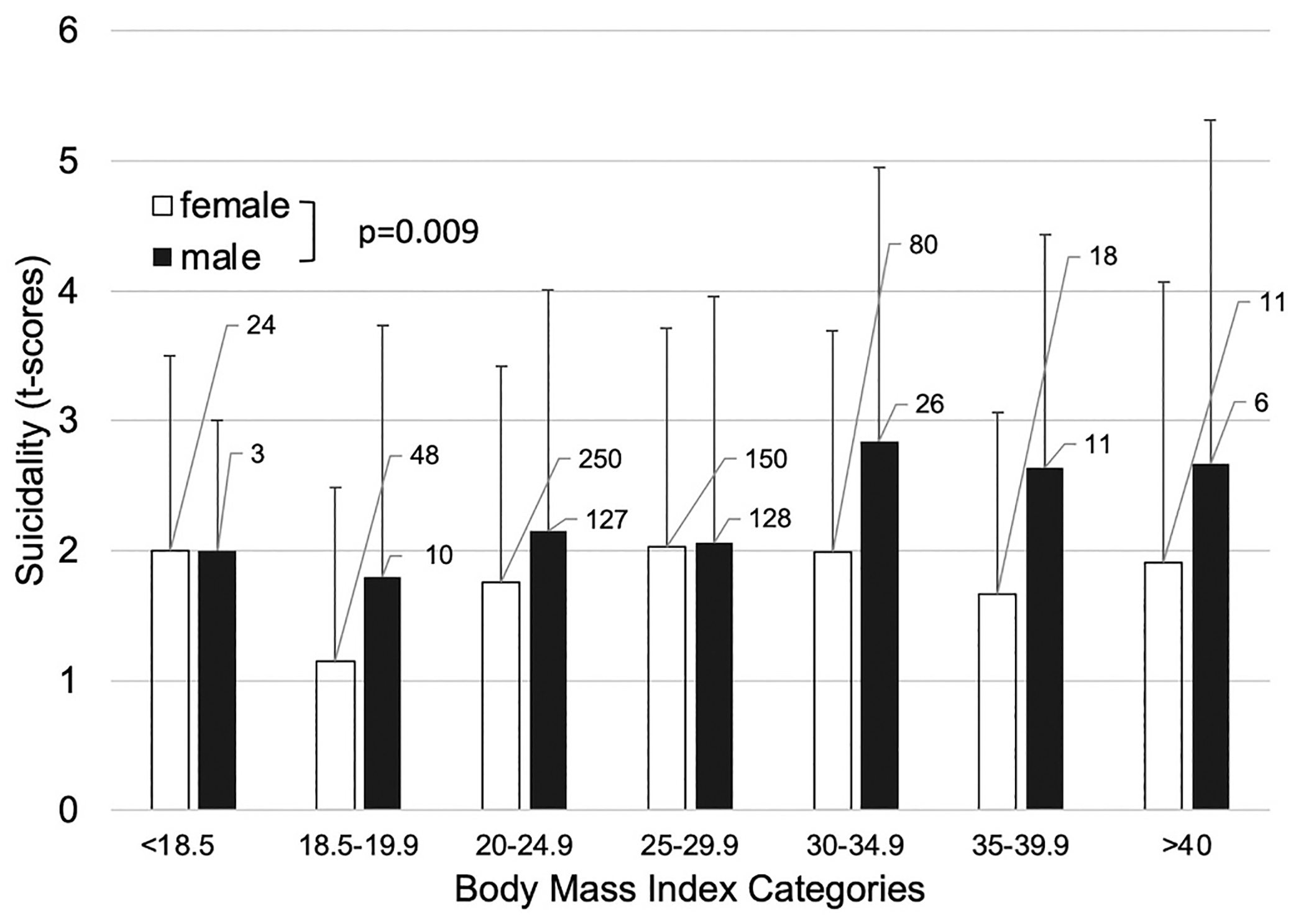
Sex differences in the baseline ‘suicidal ideation’ sub-item of the Montgomery–Åsberg Depression Rating Scale (MADRS) by body mass index (BMI) groups. The numbers next to the bars represent individuals in each category. The overall sex difference in suicidality was significant at baseline but not at the post-treatment MADRS.

MADRS subitems were analyzed to explore which psychopathological domains were associated with BMI (Fig. 2). At both baseline and post-treatment, individuals with higher BMIs showed less appetite reduction (rMADRS: F(1, 892) = 9.12, cMADRS: F(1, 892) = 20.91, both p < 0.0001) and greater impairment of concentration (rMADRS: F(1, 892) = 10.75, p = 0.002; cMADRS: F(1, 892) = 7.06, p = 0.01), regardless of treatment response. Lassitude (F(1, 892) = 5.95, p = 0.02) was also increased in higher BMI categories, but only at baseline (i.e., at the rMADRS; Fig. 3). In addition, a trend-wise association was noted between the rMADRS suicidality item and BMI, with higher BMI associated with more suicidal thoughts (F(1, 892) = 5.16, p = 0.05). Notably, there was a significant (binary) sex difference in this sample, with men with higher BMI exhibiting elevated suicidality scores compared to women with higher BMI (F(1, 892) = 8.24, p = 0.009) (see Fig. 4). Other MADRS items such as sadness, tension, sleep, inability to feel, and pessimistic thoughts were not significantly associated with BMI (all p > 0.05).

## 4. Discussion

This retrospective analysis used data from the GSRD sample to investigate the association between BMI and clinical outcomes in individuals with MDD. The most salient finding was that treatment response was not significantly associated with BMI, although there was a statistical trend for treatment resistance to be linked to overweight. However, elevated BMI was significantly associated with longer duration of psychiatric hospitalizations over their lifetimes, earlier age of MDD onset, greater number of psychiatric comorbidities, and higher suicidality—especially in obese males. These results were corrected for a risk score representing weight change due to psychopharmacotherapy. Almost half of the individuals in our sample were overweight or obese (48 %)—a rate similar to that seen in the general European population (Eurostat, 2021)—and almost a third of the sample had not responded to at least two antidepressant trials during their current MDE.

Previous evidence found bidirectional links between elevated BMI and MDD (Milaneschi et al., 2019), with the presence of one increasing the likelihood of the other. In addition, a previous controlled clinical trial found that elevated BMI mediated worse outcomes in a medication-selective manner affecting nortriptyline but not escitalopram (Uher et al., 2009). Another study found that elevated BMI significantly blunted response to selective serotonin reuptake inhibitors (SSRIs), especially in obese men (Khan et al., 2007). Furthermore, Kloiber and colleagues found that overweight or obese individuals take longer to respond to antidepressants (Kloiber et al., 2007). In this context, it is interesting to note that our calculated risk score characterizing the individual risk of psychopharmacotherapy-related weight gain was robustly associated with treatment resistance. However, a causal relationship between psychopharmacotherapy with weight gain properties, gained weight, and worse outcomes cannot be inferred from the present dataset because, theoretically, patients with higher clinical severity would receive more complex treatment, including combination and augmentation strategies in general. The results, however, suggest that close attention should be paid to how clinical trial medications may affect weight in individuals with treatment-resistant depression. The possibility of underdosing in patients with elevated BMI might be a potential cause of apparent treatment resistance, though these data were not examined in this study. On the other hand, obese patients may need higher doses to reach sufficient plasma levels for antidepressant effects. Accordingly, the results of this study indicate that body weight and weight gain should be monitored closely during antidepressant treatment.

Although the current study provided no clear evidence that elevated BMI negatively impacted treatment response in MDD, this result contrasts with previous findings with random forest classification from the GSRD database, where BMI ranked among the top variables for distinguishing treatment response from treatment resistance (Kautzky et al., 2018). This discrepancy is most likely due to methodological differences such as the statistical methods employed and patient sampling. In the present study, individuals with higher BMIs nevertheless exhibited significantly longer duration of psychiatric hospitalizations over their lifetimes, which may suggest slower convalescence despite the fact that no significant association was observed between BMI and length of the current MDE. Furthermore, a statistical trend was noted between increased BMI and higher incidence of MDEs, suggesting that not only convalescence but also stable remission may be affected by overweight. This finding differs from the GENDEP results (Uher et al., 2009), which found no association between baseline BMI and number of MDEs. In addition, our sociodemographic analysis found that overweight and obese individuals with MDD had an earlier age of onset, a tendency that may be attributable to pro-inflammatory mediators such as C-reactive protein (CRP) and interleukin-6 (IL-6), which are synthesized in response to proliferating adipose tissue (Tilg and Moschen, 2006). On the other hand, it is possible that participants with earlieronset MDD spent more of their lifetimes inactive, which may have made them more likely to develop elevated BMI. For instance, a recent analysis investigating metabolic inflammation and depression identified a statistically robust relationship between inflammatory markers and a defined set of depressive symptoms such as changes in appetite, loss of energy, and sleep problems (Frank et al., 2021). Interestingly, the effect of body weight on psychiatric disease does not seem to be restricted to MDD, as our data found that individuals with higher BMIs were more likely to suffer from some comorbid phobias or PTSD. Taken together, the evidence suggests that although only trend-wise outcomes for an association between TRD and BMI were observed, the findings nevertheless support the close clinical monitoring of weight in individuals with depression in general, and in those with overweight and obesity in particular. Indeed, a recent article found that physical exercise had beneficial effects in individuals with MDD, and this might easily be included in daily clinical practice (D’Angelantonio et al., 2022).

Notably, our results provide further evidence that suicidality is increased among overweight and obese individuals with MDD (Brunner et al., 2006; Gomes et al., 2010). The picture is less clear in nondepressed adults. For instance, in a survey of 41,598 individuals in the United States, extreme overweight (BMI ≥ 40) was associated with suicide attempts (Dong et al., 2006). In contrast, a more recent, large meta-analysis reported an increased risk of completed suicide in those with lower BMIs but lower risk in individuals with overweight and obesity (Perera et al., 2016), although the authors underscored the limited quality of the current evidence for obese patients. While the methodology of our study was based exclusively on the use of a single dichotomous variable and the MADRS sub-item ‘suicidal ideation’, suicidality nevertheless appeared to be increased in obese and overweight individuals with MDD in this sample. Furthermore, our data also identified a potential role of sex in the association between BMI and suicidality, with men exhibiting elevated scores on the ‘suicidal ideation’ item of the MADRS; it should be noted that, at the time that these data were collected, sex was still coded as binary. Although lipid profiles were not collected in this study, it is noteworthy in this context that previous meta-analyses found that lower total cholesterol levels were associated with elevated risk for suicidality in patients with MDD (Li et al., 2020; Wu et al., 2016).

Results of the exploratory analysis of MADRS sub-items should be considered preliminary, given that the MADRS was not designed to capture these dimensions in stand-alone analyses. Nevertheless, this approach can provide an initial impression of which psychopathological dimensions might be most affected by BMI in individuals with MDD. Our analysis found that reduced appetite was blunted in individuals with higher BMIs, and this did not change after antidepressant treatment. This echoes a recent genetic finding with Mendelian randomization that demonstrated a causal role of obesity on increased appetite in individuals with depression (Pistis et al., 2021). Consistent with these findings, other studies have suggested that depressed individuals with low BMIs (lower than 18.5 kg/m^2^) suffered the highest appetite reduction, and that severity of appetite reduction decreased linearly with increasing BMI (Berlin and Lavergne, 2003).

In this study, concentration was also significantly reduced in conjunction with higher BMI at retrospective baseline, as well as post-treatment. A previous study that assessed cognitive speed in MDD, as well as selective and divided attention at admission and discharge, found that individuals with normal BMIs had greater improvement in divided attention after antidepressant treatment; conversely, individuals with higher BMIs displayed significantly less improvement in selective attention at discharge than those with normal BMIs (Kloiber et al., 2007). In contrast, Uher and colleagues found no such link between BMI and cognitive or mood symptoms (Uher et al., 2009), and the characteristics of their sample—including the distribution of patients with overweight and obesity and average BMI—was remarkably similar to ours. The reasons for this discrepancy remain unclear.

The MADRS item “lassitude” was also found to be reduced in individuals with higher BMIs at baseline. To the best of our knowledge, no previous studies have explored this link. It should be noted that no significant link between reduced sleep and BMI was noted in this sample. In contrast, Kloiber and colleagues found that depressed individuals with higher BMIs had significantly lower scores on a non-atypical subscale comprising the Hamilton Rating Scale for Depression (HAM-D) (Hamilton, 1960) items of weight loss, insomnia, and loss of appetite (Kloiber et al., 2007). Uher and colleagues similarly found that higher BMI at baseline was associated with less improvement in neurovegetative symptoms such as disturbed sleep and loss of appetite (Uher et al., 2009). Finally, no significant correlation was found between BMI and the MADRS items “Apparent or Reported Sadness”, “Inner Tension”, or “Pessimistic Thoughts”. In contrast, Berlin and colleagues observed that higher BMI was associated with lower scores on the latter item (Berlin and Lavergne, 2003).

Despite these intriguing results, the study is associated with several potential limitations. First, data obtained from tertiary care and university centers were analyzed in a retrospective cross-sectional design; future studies may want to consider the advantages of longitudinal design for relating clinical variables with weight changes and include primary and secondary care centers. Second, the study measures were not designed to assess overweight and obesity; in this context, more accurate measurements of abdominal body fat such as waist-to-hip ratio or body composition measurements would be preferable for quantifying body fat. Ideally, these measures would be available over the time course of episodes of MDD and remitted states to further discern temporal dynamics between depressive states and body weight. Third, the sample was composed almost entirely of individuals of Caucasian descent (96 %) (Dold et al., 2016), which impairs generalizability to other regions. Finally, the analyses were not corrected for smoking status, though nicotine is a known appetite suppressant. The major strength of the study is the real-world conditions of MDD patients experiencing a wide range of disease course, symptom severity, and clinical manifestations—including suicidality and comorbidities—who were treated in inpatient or outpatient settings at university as well as non-academic centers. This differentiates the present study from most randomized, controlled trials that use stringent inclusion criteria that do not enable such challenging clinical heterogeneity.

## 5. Conclusions

This retrospective analysis of the multicenter GSRD data found that overweight and obese individuals with MDD were at higher risk for suicidality and required longer psychiatric inpatient stays. A trend-wise association between higher BMI and treatment-resistant depression was also observed. These findings suggest that obese and overweight individuals with MDD should be closely monitored—as should weight gain in individuals with MDD in general—in order to prevent negative psychiatric and somatic medical outcomes.

## Figures and Tables

**Table 1:** Statistical results.

	Predictor	Estimate	Std. error	t-Value/z-value	p-Value
*A: Treatment outcome, BMI*					
Response/TRD	Risk score	0.285	0.064	4.435	<0.0001**
	BMI	0.025	0.014	1.725	*0.085*
*B: Clinical and sociodemographic parameters*					
Suicidality (yes/no)	BMI	10.042	0.014	3.048	0.002*
	Risk Score	0.186	0.057	3.268	0.001*
Hospitalization time (weeks)	BMI	0.18	0.065	2.776	0.006*
	Age	0.078	0.023	3.456	0.0006**
	Risk Score	1.669	0.267	6.244	<0.0001**
Age of first episode (years)	BMI	0.206	0.081	−2.547	0.011*
	Sex	1.87	0.854	2.19	0.029*
Number of MDEs	BMI	0.037	0.021	1.751	*0.08*
	Sex	−0.493	0.225	−2.192	0.029*
	Age	0.019	0.007	2.64	0.008*
Duration of MDE (weeks)	BMI	0.251	1.475	0.17	n.s.
Agoraphobia (yes/no)	BMI	0.069	0.025	2.723	0.006*
Social phobia (yes/no)	BMI	0.069	0.033	2.103	0.035*
PTSD (yes/no)	BMI	0.135	0.043	3.138	0.002*
	Age	−0.06	0.026	−2.335	0.02*
GAD (yes/no)	BMI	−0.005	0.022	−0.221	n.s.
Panic disorder (yes/no)	BMI	0.032	0.022	1.426	n.s.
OCD (yes/no)	BMI	0.029	0.067	0.434	n.s.
Psychotic symptoms (yes/no)	BMI	−0.006	0.032	−0.2	n.s.
Dysthymia (yes/no)	BMI	0.066	0.096	0.692	n.s.
*C: MADRS items*					
# 1 Apparent Sadness, baseline	BMI	−0.002	0.007	−0.224	n.s.
# 1 Apparent Sadness, post-treatment	BMI	0.004	0.011	0.316	n.s.
# 2 Reported Sadness, baseline	BMI	0.002	0.006	0.381	n.s.
# 2 Reported Sadness, post-treatment	BMI	0.008	0.011	0.736	n.s.
# 3 Inner Tension, baseline	BMI	−0.011	0.007	−1.483	n.s.
# 3 Inner Tension, post-treatment	BMI	0.004	0.009	0.445	n.s.
# 4 Reduced Sleep, baseline	BMI	0.008	0.009	0.879	n.s.
# 4 Reduced Sleep, post-treatment	BMI	0.015	0.0116	1.261	n.s.
# 5 Appetite Reduction, baseline	BMI	−0.04	0.0114	−3.484	0.0005**
	Age	0.022	0.004	5.368	<0.0001**
	Risk Score	0.096	0.047	2.06	0.04
# 5 Appetite Reduction, post-treatment	BMI	−0.048	0.01	−4.963	<0.0001**
	Age	0.012	0.003	3.569	0.0003**
	Risk Score	0.19	0.039	4.802	<0.0001**
# 6 Concentration, baseline	BMI	0.022	0.007	3.046	0.002*
	Risk Score	0.096	0.0297	3.225	0.001*
# 6 Concentration, post-treatment	BMI	0.023	0.01	2.44	0.015*
	Risk score	0.171	0.04	4.314	<0.0001**
# 7 Lassitude, baseline	BMI	0.016	0.007	2.322	0.02*
	Age	0.005	0.002	1.984	0.048*
# 7 Lassitude, post-treatment	BMI	0.017	0.011	1.567	n.s.
# 8 Inability to Feel, baseline	BMI	0.012	0.008	1.589	n.s.
# 8 Inability to Feel, post-treatment	BMI	0.017	0.011	1.623	n.s.
# 9 Pessimistic Thoughts, baseline	BMI	−0.012	0.008	−1.524	n.s.
# 9 Pessimistic Thoughts, post-treatment	BMI	0.003	0.01	0.292	n.s.
# 10 Suicidal Ideation, baseline	BMI	0.023	0.012	1.964	0.05
	Sex	0.32	0.122	2.628	0.009*
	Risk score	0.217	0.05	4.594	<0.0001**
# 10 Suicidal Ideation, post-treatment	BMI	0.012	0.009	1.324	n.s.

A. Treatment outcome and BMI according to response status; non-response (i.e., treatment-resistant depression) was defined as insufficient reduction in MADRS score after two adequate antidepressant treatment trials. B. Clinical and sociodemographic parameters and their relationship to BMI controlled for sex, age, and weight gain risk due to medication. Variables were obtained from case report forms. Only significant results of covariates are shown. C. Exploratory MADRS sub-item analyses at retrospective baseline and post-treatment. The risk score represents risk of weight gain according to medication.

Abbreviations: BMI: body mass index; GAD: generalized anxiety disorder; MADRS: Montgomery–Åsberg Depression Rating Scale; MDE: major depressive episode; OCD: obsessive-compulsive disorder; PTSD: post-traumatic stress disorder; TRD: treatment resistant depression.

* p < 0.05 uncorrected.

** Bonferroni corrected p-values.
